# Point-of-care detection of *Neisseria gonorrhoeae* based on RPA-CRISPR/Cas12a

**DOI:** 10.1186/s13568-023-01554-7

**Published:** 2023-05-27

**Authors:** Qianrong Tu, Xiaoying Cao, Chao Ling, Lili Xiang, Ping Yang, Shifeng Huang

**Affiliations:** 1 Department of Clinical Laboratory Medicine, The First Affiliated Hospital of Chongqing Medical University, No. 1 Friendship Road, Yuzhong District, Chongqing, 400016 People’s Republic of China; 2 Department of Burn and Plastic Surgery, The First Affiliated Hospital of Chongqing Medical University, No. 1 Friendship Road, Yuzhong District, Chongqing, 400016 People’s Republic of China; 3 Department of Clinical Laboratory Medicine, Chongqing Shapingba District Chenjiaqiao Hospital, Chongqing, 401331 People’s Republic of China

**Keywords:** *Neisseria gonorrhoeae*, Recombinase polymerase amplification, CRISPR/Cas12a, Fluorescence, Lateral flow detection

## Abstract

Gonorrhea, caused by *Neisseria gonorrhoeae* (*N. gonorrhoeae*), is a persistent global public health threat. The development of low-cost, point-of-care testing is crucial for gonorrhea control, especially in regions with limited medical facilities. In this study, we integrated CRISPR/Cas12a reaction with recombinase polymerase amplification (RPA) to provide a simple and adaptable molecular detection method for *N. gonorrhoeae*. The RPA-Cas12a-based detection system developed in this study enables rapid detection of *N. gonorrhoeae* within 1 h without the use of specialized equipment. This method is highly specific for identifying *N. gonorrhoeae* without cross-reactivity with other prevalent pathogens. Furthermore, in the evaluation of 24 clinical samples, the detection system demonstrates a 100% concordance rate with traditional culture, which is being used clinically as a reference method. Overall, the RPA-Cas12a-based *N. gonorrhoeae* detection has the advantages of rapidity, portability, low-cost, no special equipment required, and strong operability, and has a high potential for application as a self-testing and point-of-care diagnosis, which is critical for the clinical management of gonorrhea in developing countries lacking medical equipment.

**Graphical Abstract:**

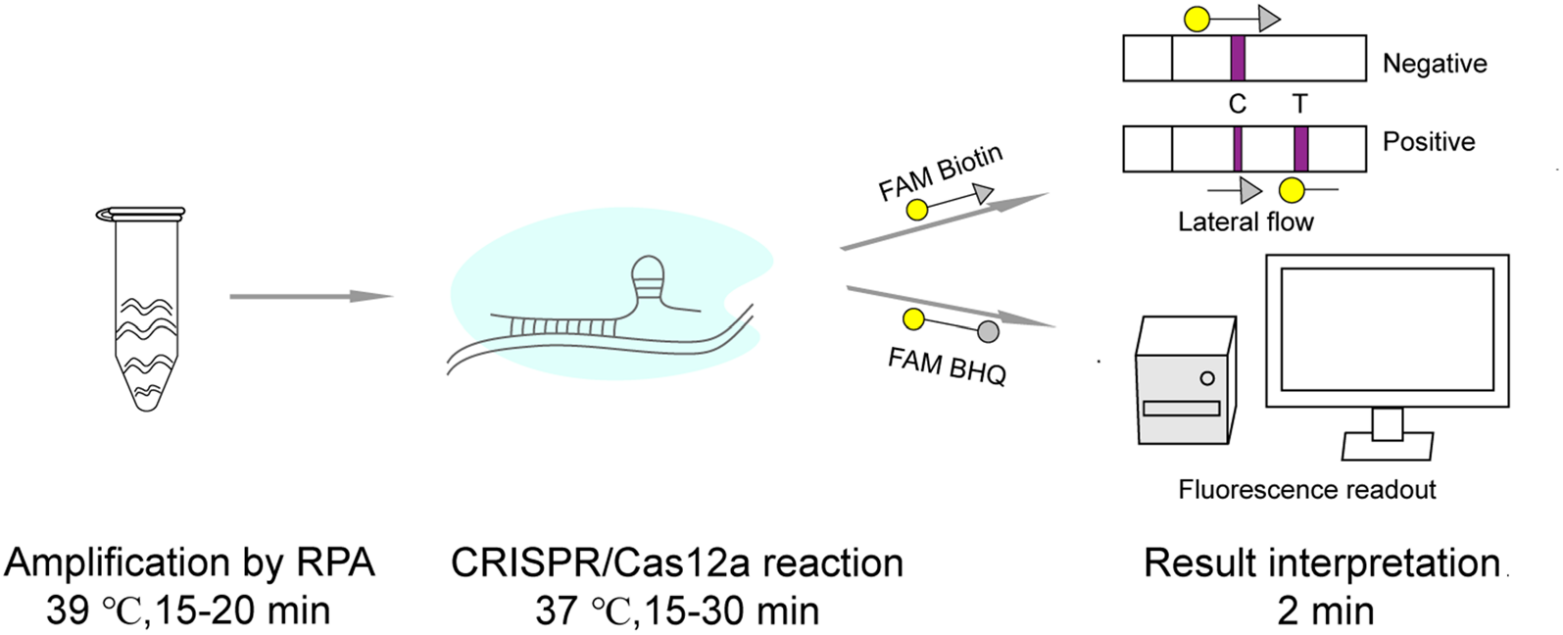

**Supplementary Information:**

The online version contains supplementary material available at 10.1186/s13568-023-01554-7.

## Key points

- Rapid detection of N. gonorrhoeae based on CRISPR/Cas12a and RPA.
- Specific and sensitive detection.
- No special equipment promises applications for point-of-care diagnosis.

**Supplementary Information:**

The online version contains supplementary material available at 10.1186/s13568-023-01554-7.

## Introduction

Gonorrhea is a sexually transmitted infection (STI) caused by the bacterium *Neisseria gonorrhoeae* (*N. gonorrhoeae*), with purulent inflammation of the urogenital tract as the main clinical symptom (Lovett and Duncan 2018; Stevens and Criss 2018). Moreover, gonorrhea is associated with infertility, adverse pregnancy and newborn outcomes (Stevens and Criss 2018; Vallely et al. 2021), and it facilitates HIV acquisition and transmission (Stenger et al. 2021). Infrequently, *N. gonorrhoeae* infection can spread systemically, causing septic arthritis, skin manifestations (Lovett and Duncan 2018), and septicemia (WHO 2016). Gonorrhea is a global public health challenge. According to the World Health Organization (WHO), the annual incidence of gonorrhea cases among adults (15–49 years old) was estimated at 82.4 million globally in 2020 (WHO 2021), with the highest prevalence in low-income countries (Rowley et al. 2019). With a rapidly rising incidence (Qin et al. 2019; Yin et al. 2018), gonorrhea has become one of the most frequently reported infectious diseases in China (Yin et al. 2018). Nationwide, the detection rate of *N. gonorrhoeae* is quite low despite its considerable prevalence. Traditional methods for *N. gonorrheae* identification mainly include culture and direct microscopy. Culture is labor-intensive and time-consuming; direct microscopy necessitates extensive laboratory technician experience and is prone to misdiagnosis. The PCR technique necessitates sophisticated equipment that may not be readily available in hospitals with minimal resources. Therefore, it is crucial to create a sensitive, low-cost, and rapid point-of-care test for *N gonorrhoeae*.

Recent advancements in clustered regularly interspaced short palindromic repeats (CRISPR)/associated proteins (Cas) systems offer a new exciting and promising avenue for molecular diagnostics. On the basis of CRISPR/Cas systems, researchers have successfully developed a variety of sensitive, specific and rapid detection platforms for detecting various pathogens, such as SHERLOCK (Cas13a) (Gootenberg et al. 2017), DETECTR (Cas12a) (Chen et al. 2018), and Cas14-DETECTR (Harrington et al. 2018). Cas12a (Cpf1), an RNA-guided endonuclease, has both cis- and trans-cleavage DNase activities (Zetsche et al. 2015; Swarts 2019). After targeted recognition of DNA sequences complementary to CRISPR RNA (crRNA), a cis-cleavage activity is displayed by Cas12a, which cleaves double-stranded DNA (dsDNA) (Swarts 2019). Subsequently, the activated Cas12a displays collateral trans-cleavage activity, that is, nonspecific cleavage of nearby untargeted single-stranded DNA (ssDNA), an activity extensively explored for use in nucleic acid diagnostics (Swarts 2019). By introducing fluorophore and quencher-labeled or fluorophore and biotin-labeled ssDNA into the reaction system, CRISPR/Cas12a cleavage results can be detected by fluorescence readout or lateral flow test strips (Xiong et al. 2020).

The isothermal amplification feature of recombinase polymerase amplification (RPA) reduces the equipment needs and opens up new avenues for breaking past laboratory boundaries. In addition, RPA’s tolerance to background DNA and certain (PCR) inhibitors such as haemoglobin, heparin, and urine (Li et al. 2018), which facilitates its clinical application, is of great significance.

The *porA* pseudogene was often selected as the target for *N. gonorrhoeae* detection due to its high specificity (Mangold et al. 2007). The *porA* gene of *N. meningitidis* encoded porin protein, while the *porA* pseudogene was not expressed in *N. gonorrhoeae*. As a result of the absence of positive selection pressure, the pseudogene appears to be extremely stable and has remained nearly unchanged over time (Hjelmevoll et al. 2006). In addition, the *porA* gene/pseudogene was found only in two human-pathogenic *Neisseria* species, *N. meningitidis* and *N. gonorrhoeae* (Hjelmevoll et al. 2006). Importantly, the *porA* pseudogene of *N. gonorrhoeae* to be identified is sufficiently distinct from the *porA* gene of *N. meningitidis* (Hjelmevoll et al. 2006).

Based on the DETECTER platform, this study has integrated CRISPR/Cas12a reaction with RPA to establish a new nucleic acid diagnositic method for *N. gonorrhoeae*. The RPA-Cas12a system enables rapid detection of *N. gonorrhoeae* without the need for specialized equipment, which is of great significance for the diagnosis and control of gonorrhea in developing countries lacking medical equipment.

## Materials and methods

### Reagents

Primers for RPA were synthesized by TsingKe Biological Technology (Beijing, China). Recombinant RNase inhibitor, crRNA, and RNase-free water were obtained from Takara Bio (Beijing, China). The ssDNA reporter was synthesized by Sangon Biotech (Shanghai, China). The TIANamp Bacteria DNA kit was purchased from TIANGEN (Beijing, China). The TwistAmp Basic Kit for RPA reaction was purchased from TwistDx (Cambridge, UK). Lba Cas12a (Cpf1) and NEBuffer r2.1 were purchased from New England Biolabs (Beijing, China). Milenia HybriDetect for lateral flow detection was purchased from Milenia Biotec (Gießen, Germany).

### Bacterial strain and clinical samples

*N. gonorrhoeae* reference strain (ATCC 43,069) was donated by Professor Xiaobing Zhang. *N. gonorrhoeae* clinical isolates (n = 8) were identified by Gram staining and mass spectrometry. 10 non-*N. gonorrhoeae* clinical isolates, including *Neisseria sicca* (*N. sicca,* n = 2), *N. meningitidis* (n = 2), *Ureaplasma urealyticum* (*U. urealyticum*, n = 1), *Pseudomonas aeruginosa* (*P. aeruginosa*, n = 1), *Enterobacter cloacae* (*E. cloacae*, n = 1), *Klebsiella pneumoniae* (*K. pneumoniae*, n = 1), *Enterococcus* *faecium* (*E.* *faecium*, n = 1), and *Acinetobacter radioresistens* (*A. radioresistens*, n = 1), were selected as negative controls. In addition, 24 clinical samples were collected from patients suspected of having gonorrhea, including cervical or vaginal discharge from women and urethral secretions from men. Sufficient clinical samples were manually separated into two equal halves, one for traditional culture (gold standard) and one for DNA extraction for molecular diagnosis using the RPA-Cas12a system. All the clinical isolates and samples described above were obtained from the First Affiliated Hospital of Chongqing Medical University.

### RPA primers, crRNA and ssDNA reporter design

All the available *porA* pseudogene sequences of *N. gonorrhoeae* (Accession Numbers AJ010732.1, AJ010733.1, AJ223449.1, AJ223448.1, AJ223447.1, and AJ223446.1) were downloaded from the NCBI database (www.ncbi.nlm.nih.gov) and aligned by Mega-X (www.megasoftware.net) to identify the conserved regions. To distinguish *N. gonorrhoeae* from *N. meningitidis*, we aligned the conserved sequences of the *porA* pseudogene of *N. gonorrhoeae* with the *porA* gene of 228 *N. meningitidis* strains (the NCBI accession numbers of *porA* gene of *N. meningitidis* were listed in Additional file 1: Table S1). RPA primers (Additional file 1: Table S2) were designed in accordance with the TwistDx instruction manual (www.twistdx.co.uk) to cover the conserved *porA* pseudogene region that differs significantly from the *porA* gene sequences of *N. meningitidis*. IDT OligoAnalyzer (www.idtdna.com) (Kersting et al. 2014) was used to examine the primer dimer and hairpin structures. The primers’ specificities were verified by NCBI’s Primer-BLAST (https://www.ncbi.nlm.nih.gov/tools/ primer-blast). We designed crRNA (Additional file 1: Table S3) for Cas12a to recognize a 21-bp target sequence near the PAM site (TTTN, N = A/C/G) in the amplicon between RPA primer pairs, and checked the crRNA sequence using the BLAST algorithm. Fluorescent ssDNA reporter was labeled by fluorophore and quencher (Additional file 1: Table S4). Lateral flow ssDNA reporter was labeled by fluorophore and biotin (Additional file 1: Table S4).

### Nucleic acid preparation

Bacterial genomic DNA was extracted with the TIANamp Bacteria DNA kit per the manufacturer's instructions. The DNA concentration ranged from 20 ng/µL to 100 ng/µL, and was stored at – 20 ℃ before use after being determined using a NanoDrop 2000 (Thermo, United States). For sensitivity testing, a solution containing genomic DNA from a *N. gonorrhoeae* reference strain was diluted to various concentrations with RNase free water.

### The RPA reaction

To achieve robust assay performance and a specific RPA reaction, a comparative evaluation of the specificity of multiple candidate RPA primer pairs in identifying *N. gonorrhoeae* was carried out. Briefly, six candidate primer pairs were used to amplify *N. gonorrhoeae* and non-*N. gonorrhoeae* strain sequences, and the optimal primer pairs were evaluated by nucleic acid electrophoresis or the CRISPR/Cas12a-fluorescent assay. Using a commercial RPA kit, the RPA reaction was carried out at 39 ℃ for 15–20 min (Table 1) according to the instructions.

**Table 1:** The RPA reaction system

Component	Volume (µL)
Primer F (10 µM)	2.4
Primer R (10 µM)	2.4
Primer-free rehydration buffer	29.5
RNase free water	11.2
Template	2

Add the above reaction mixure to a tube containing lyophilized RPA enzyme. Then add 2.5 µL 280 mM MgOAc before incubation at 39 ℃ for 15–20 min

### RPA-Cas12a-fluorescent assay

Fluorophore and quencher-labeled ssDNA reporter was introduced into the Cas12a-fluorescent system. Following the RPA reaction, the RPA product was added as substrate into the Cas12a-fluorescent system. The RPA-Cas12a-fluorescent reaction was carried out at 37 °C in a final volume of 20 µL (as specified in Table 2). The fluorescence signal was examined by TECAN infinite 200Pro (TECAN, Switzerland) with an excitation wavelength of 492 nm, and an emission wavelength of 522 nm. The optimization of cleavage time within the range of 5 to 55 min was undertaken to attain desirable performance of the CRISPR/cas12a system.

**Table 2:** The RPA-Cas12a assay system

Component	Volume (µL)
NEBuffer r2.1 (10 ×)	2
Cas12a (1 µM)	1
crRNA (1 µM)	1.2
Fluorescent ssDNA reporter (2 µM), or Lateral flow ssDNA reporter (5 µM)	1
RNase inhibitor RNase free water RPA products	0.25 13.55 1

### RPA-Cas12a-LFD assay

Except for the ssDNA reporter, the RPA-Cas12a-lateral flow detection (LFD) system contained the same components as the RPA-Cas12a-fluorescent assay (Table 2). In addition, after CRISPR/Cas12a cleavage at 37 °C, the CRISPR/Cas12a reaction product was diluted proportionately in Hybri Detect Assay Buffer to meet the solution volume requirements for LFD. The strips were then inserted and incubated at room temperature for 2 min before being withdrawn and photographed. ImageJ (https://imagej.nih.gov/ij/) quantification and GraphPad (www.graphpad.com) visualization were used to examine the band intensities of the test line of the strips. Several parameters of the RPA-Cas12a-LFD system were optimized, such as the ssDNA concentration (3.125 nmol/L, 6.25 nmol/L, 12.5 nmol/L, 25 nmol/L, 62.5 nmol/L, 125 nmol/L, 250 nmol/L), the volume ratio of Cas12a cleavage product to Hybri Detect Assay Buffer (1:1, 1:3). Commercial strips were used for lateral flow detection. The Milenia HybriDetect kit contains the Hybri Detect Assay Buffer and lateral flow strips.

### Specificity and sensitivity of the RPA-Cas12a system

Using DNA templates from the *N. gonorrhoeae* reference strain (ATCC43069), eight *N. gonorrhoeae* clinical isolates and ten non-*N. gonorrhoeae* clinical isolates (including *N. sicca*, *N. meningitidis*, *U. urealyticum*, *P. aeruginosa*, *E. cloacae*, *K. pneumoniae*, *E.* *faecium*, *A. radioresisten*s), the specificity of the RPA-Cas12a system was evaluated. Following a 20-min RPA reaction, the RPA products were added into both the Cas12a-fluorescent system and the Cas12a-LFD system.

The sensitivity of the RPA-Cas12a system was evaluated using serial dilutions of genomic DNA of the *N. gonorrhoeae* reference strain (500 pg/µL, 50 pg/µL, 5 pg/µL, 500 fg/µL, 50 fg/µL, 5 fg/µL). The RPA products were added into both the Cas12a-fluorescent system and the Cas12a-LFD system following a 20-min RPA reaction.

### Clinical validation of the RPA-Cas12a system

The feasibility of the RPA-Cas12a system was evaluated using 24 clinical samples from individuals suspected of having gonorrhea, with traditional culture as a reference method. Sufficient clinical samples were manually separated into two equal halves, one for traditional culture, and the other for DNA extraction for molecular diagnosis using RPA-Cas12a-fluorescent and RPA-Cas12a-LFD assays. In other words, all clinical samples were evaluated using the aforementioned three methods, and the test results were compared with those of the culture method. Culturing was performed on Thayer-Martin (TM) medium in 5% CO_2_ at 37 °C for 24–72 h (Su et al. 2011).

## Results

### Construction of specific RPA reaction

**Fig. 1:**
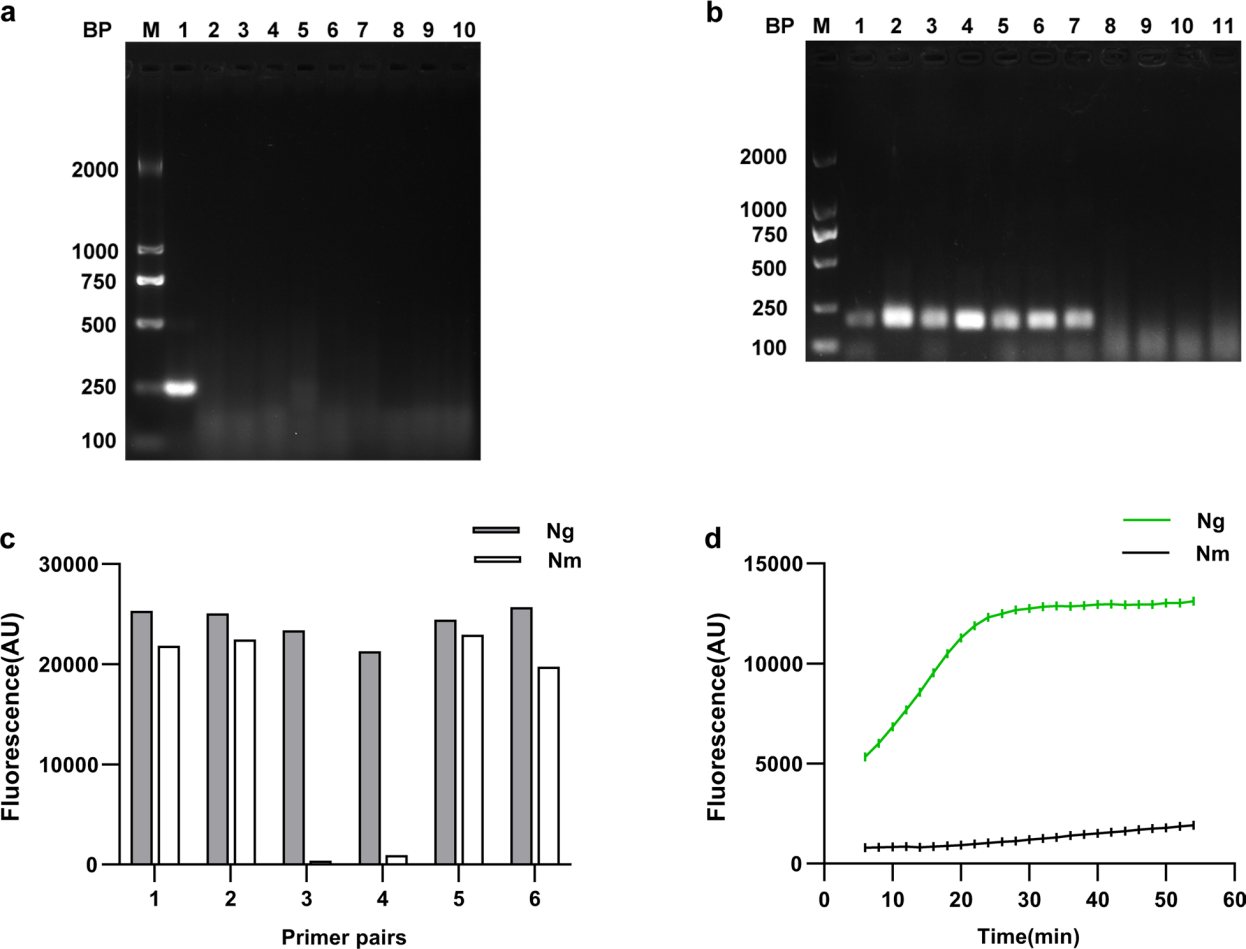
Optimizations of the RPA primer pairs and the CRISPR/Cas12a cleavage time durations. **a** Evaluation of the specificity of the RPA primer pair No.3 by electrophoresis on a 2% agarose gel. 1, *N. gonorrhoeae* ATCC43069; 2, *E.* *faecium*; 3, *N. sicca*; 4, *N. sicca*; 5, *N. meningitidis*; 6, *N. meningitidis*; 7, *P. aeruginosa*; 8, *A. radioresistens*; 9, *E. cloacae*; 10, *K. pneumoniae*.** b** Evaluation of the specificity of the RPA primer pair No.6 by electrophoresis on a 2% agarose gel. 1–7, *N. gonorrhoeae* clinical isolates; 8, *A. radioresistens*; 9, *K. pneumoniae*; 10, *N. meningitidis*; 11, *N. meningitidis*. BP, base pair; M, marker. **c** Evaluation of the specificity of the RPA primer pairs by Cas12a detection system. RPA reactions were performed with 6 sets of primer pairs. **d** Optimizations of the CRISPR/Cas12a cleavage time durations. *Ng*, *N. gonorrhoeae* ATCC43069 (1 ng/µL); *Nm*, *N. meningitidis*. AU, arbitrary unit

The specificities of the six RPA primer pairs used to pre-amplify the *porA* pseudogene of *N. gonorrhoeae* were initially validated using NCBI’s Primer-BLAST. In the process of identifying the optimal primer pairs, we discovered an intriguing phenomenon. Candidate primer pairs were used to amplify *N. gonorrhoeae* and non-*N. gonorrhoeae* strain sequences, and the nucleic acid electrophoresis results indicated that the primer pair No. 3 and No. 6 were specific (Fig. 1a, b). However, when the RPA amplification product generated by primer pair No. 6 was added to the Cas12a detection system, the specificity of the detection system was compromised. Therefore, we abandoned the direct screening of specific primer pairs by electrophoresis and instead observed the specificity in conjunction with the Cas12a-fluorescent detection system to select the most suitable RPA primer pairs for the Cas12a detection system. As depicted in Fig. 1c, the primer pair No. 3 exhibited a high degree of specificity, which is advantageous for the Cas12a detection system.

### Construction of the RPA-Cas12a-fluorescent assay

Under the condition of producing distinct fluorescence intensity, the shorter the CRISPR/Cas12a cleavage time, the quicker the RPA-Cas12a-fluorescent assay. Therefore, the cleavage time was optimized between 5 and 55 min, and because the fluorescence was consistent at 30 min, 30 min was chosen as the optimal time. However, the fluorescence intensity of *N. gonorrhoeae* was readily distinguishable from that of the negative control strain *N. meningitidis* before reaching a plateau. Thus the duration of cleavage can be reduced to 15 to 30 min (Fig. 1d).

### Construction of the RPA-Cas12a-LFD assay

**Fig. 2:**
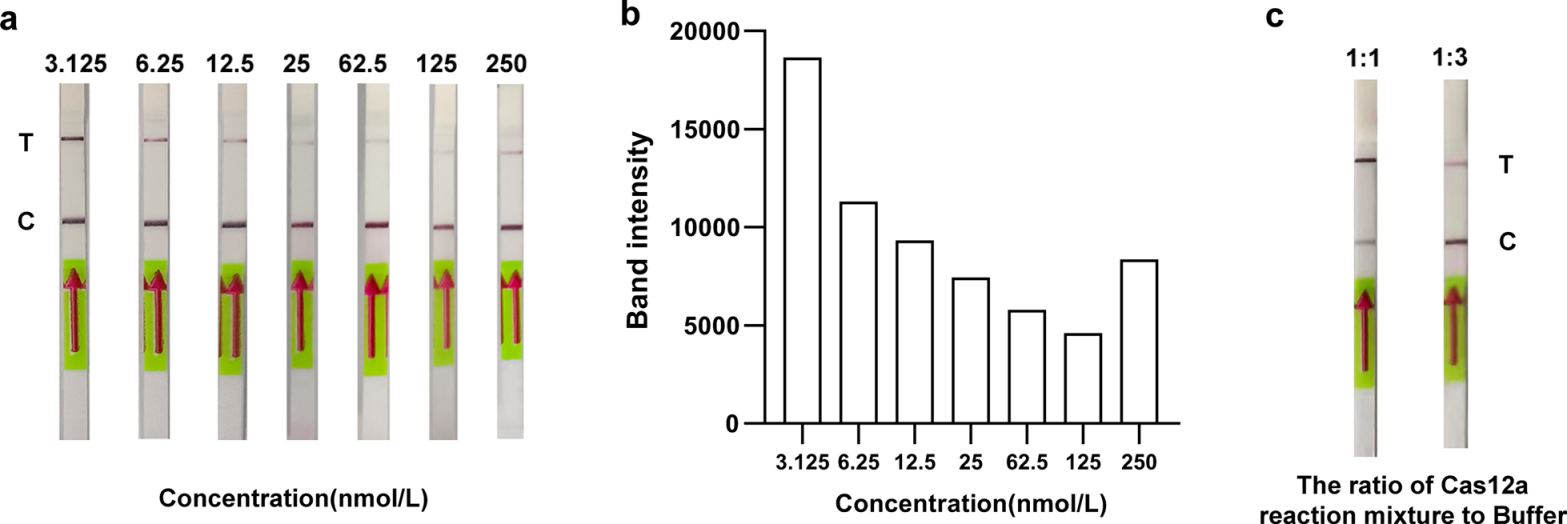
Construction of the RPA-Cas12a-LFD assay **a**, **b** The optimization of the concentration of the lateral flow detection ssDNA reporter (3.125 nmol/L, 6.25 nmol/L, 12.5 nmol/L, 25 nmol/L, 62.5 nmol/L, 125 nmol/L, 250 nmol/L). C, control line; T, test line. Band intensity, intensity of the test line was measured by ImageJ.** c** The optimization of the ratio of Cas12a reaction mixture to Hybri Detect Assay Buffer. Cas12a reaction mixture: Hybri Detect Assay Buffer (1:1, 1:3)

Although the RPA-Cas12a-fluorescent assay was easy-to-implement, it was dependent on the fluorescent readout instrument, therefore, we developed the RPA-Cas12a-LFD assay to achieve the on-site diagnosis of *N. gonorrhoeae*, with the advantages of portability, low cost and no need of specialized equipment. To reduce false-positive results in the RPA-Cas12a-LFD assay, we diluted ssDNA into different concentrations. ImageJ quantification and GraphPad visualization of the band intensities of the test line of lateral flow strips revealed that 125 nmol/L ssDNA produced the lowest band intensity for the test line of negative samples (Fig. 2a, b). In addition, the volume ratio between the Cas12a reaction mixture and the Hybri Detect Assay Buffer was also optimized. The Cas12a reaction mixture was diluted with the Hybri Detect Assay Buffer in different proportions (1:1, 1:3), and the findings demonstrated that a ratio of 1:1 can make the positive band the most prominent (Fig. 2c).

### Specificity and LoD of RPA-Cas12a system

**Fig. 3:**
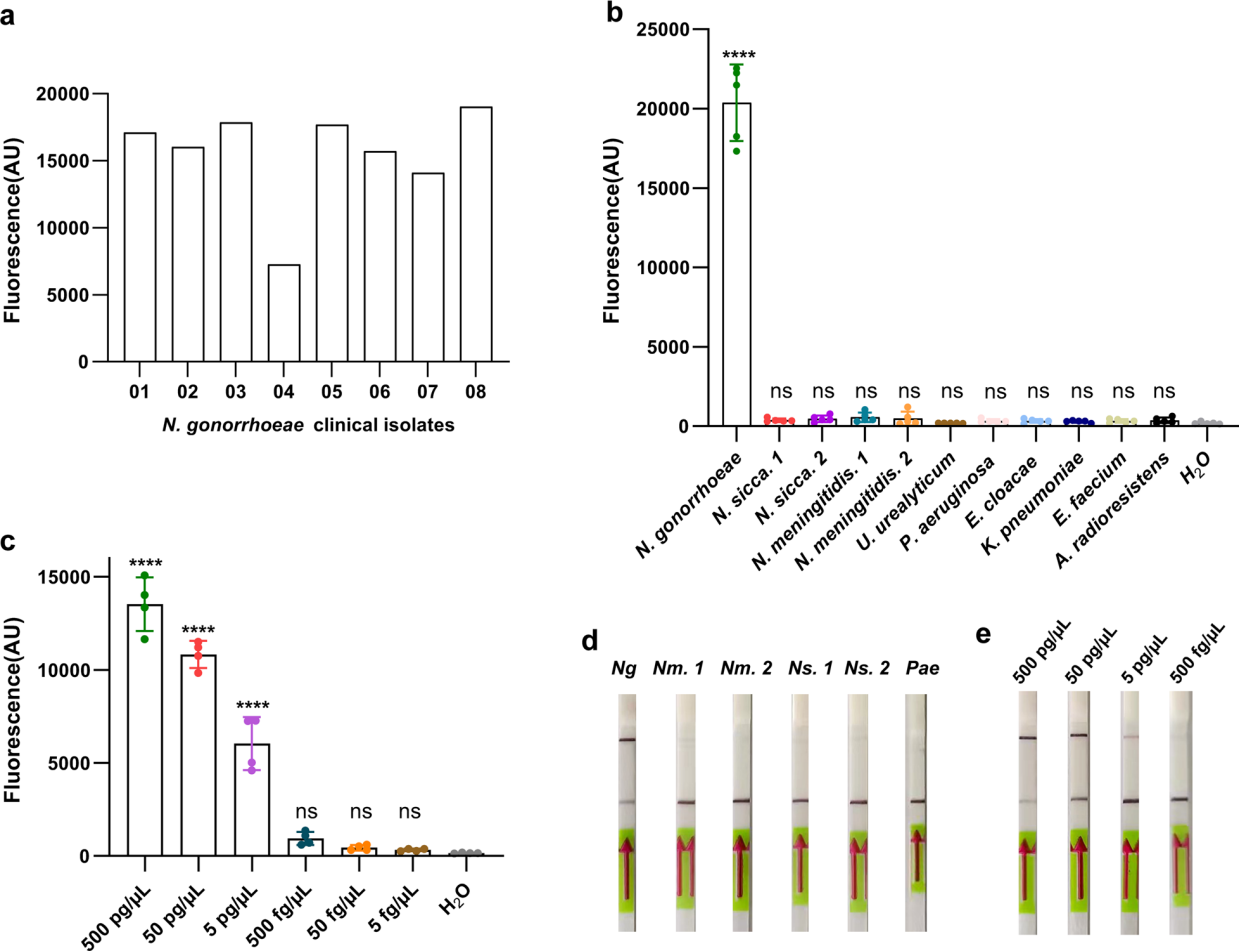
Specificity and LoD of RPA-Cas12a system **a**, **b** Specificity of RPA-Cas12a-fluorescent assay, with reaction lasting for 30 min. **c** The LoD of RPA-Cas12a-fluorescent assay, with reaction lasting for 30 min. **d** Specificity of RPA-Cas12a-LFD assay, with reaction lasting for 30 min. **e** The LoD of RPA-Cas12a-LFD assay, with reaction lasting for 30 min. Data points represent at least four biologically independent experiments. Error bars indicate the mean ± SD. AU, arbitrary unit. The asterisk indicates a significant difference compare with the control of no targets by one-way ANOVA analysis (*P < 0.05, ****P < 0.0001). ns, not statistically significant. *Ng*, *N. gonorrhoeae* ATCC43069; *Nm.* 1, *N. meningitidis*. 1; *Nm*. 2, *N. meningitidis*. 2; *Ns*. 1, *N. sicca*. 1; *Ns*. 2, *N. sicca*. 2; *Pae*, *P. aeruginosa*

DNA extracts from *N. gonorrhoeae* reference strain (ATCC43069), *N. gonorrhoeae* clinical isolates (n = 8) and non-*N. gonorrhoeae* clinical isolates (*N. sicca*, *N. meningitidis*, *U. urealyticum*, *P. aeruginosa*, *E. cloacae*, *K. pneumoniae*, *E.* *faecium*, *A. radioresistens*) were employed as templates to evaluate the specificity of the RPA-Cas12a system. Both the RPA-Cas12a-fluorescent and RPA-Cas12a-LFD assays can accurately identify *N. gonorrhoeae* without cross-reactivity with non-*N. gonorrhoeae* (Fig. 3a, b, d), indicating high specificity.

To determine the limit of detection (LoD) of the RPA-Cas12a system, serially diluted *N. gonorrhoeae* ATCC43069 DNA extracts were used as templates (500 pg/µL, 50 pg/µL, 5 pg/µL, 500 fg/µL, 50 fg/µL, 5 fg/µL). The results showed that the LoD of the RPA-Cas12a-fluorescent assay and RPA-Cas12a-LFD assay were both as low as 5 pg/μL (Fig. 3c, e), demonstrating high sensitivity.

### Clinical validation of the RPA-Cas12a system

**Fig. 4:**
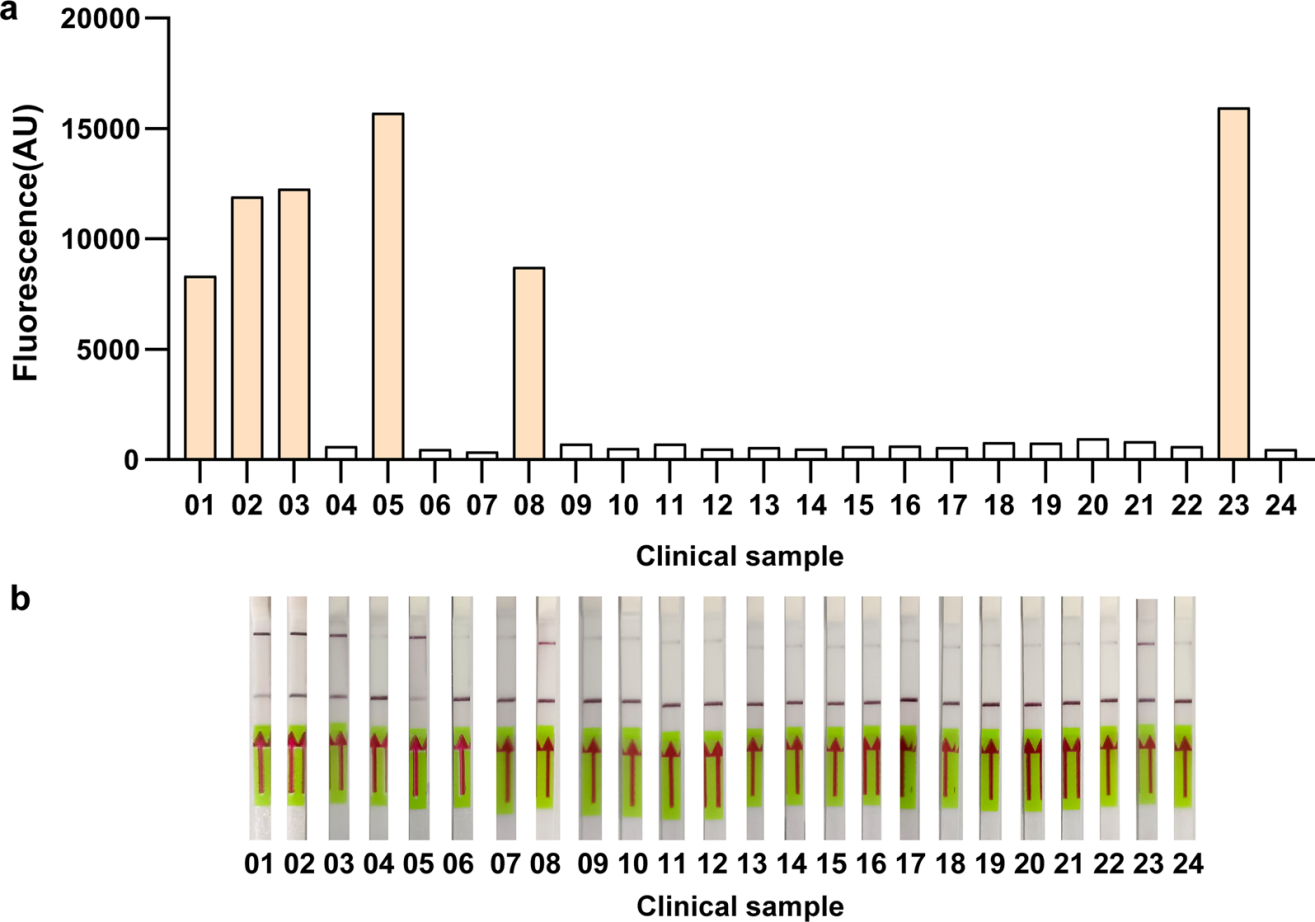
Clinical validation. **a** Clinical validation of the RPA-Cas12a-fluorescent assay. 24 clinical samples from individuals suspected of having gonorrhea were examined by the RPA-Cas12a-fluorescent assay. AU, arbitrary unit. **b** Clinical validation of the RPA-Cas12a-LFD assay. 24 clinical samples from individuals suspected of having gonorrhea were examined by the RPA-Cas12a-LFD assay

In addition, the feasibility of the RPA-Cas12a-based *N. gonorrhoeae* detection system was evaluated using clinical samples from 24 individuals suspected of having gonorrhea. When taking traditional culture as a reference method, both RPA-Cas12a-fluorescent assay and RPA-Cas12a-LFD assay demonstrated a 100 percent concordance rate (Fig. 4a, b; Table 3). Our method promises clinical applications for the identification of *N. gonorrhoeae* due to its high feasibility.

**Table 3:** Clinical validation of the RPA- Cas12a system

	Clinical samples																							
	1	2	3	4	5	6	7	8	9	10	11	12	13	14	15	16	17	18	19	20	21	22	23	24
RPA-Cas12a fluorescent assay	+ ^a^	+	+	−^b^	+	−	−	+	−	−	−	−	−	−	−	−	−	−	−	−	−	−	+	−
RPA-Cas12a LFD assay	+	+	+	−	+	−	−	+	−	−	−	−	−	−	−	−	−	−	−	−	−	−	+	−
Traditional culture	+	+	+	−	+	−	−	+	−	−	−	−	−	−	−	−	−	−	−	−	−	−	+	−

^a^N. gonorrhoeae was detected.

^b^*N. gonorrhoeae* was not detected

## Discussion

Current clinical methods for detecting *N. gonorrhoeae*, whether the traditional culture, direct microscopy, or the emerging PCR method, all require specialized equipments and skilled technicians, limiting the convenience and adaptability of the detection. The RPA-Cas12a-based *N. gonorrhoeae* detection system developed in this study provides rapid detection of *N. gonorrhoeae* without the need for specialized equipment and has significant potential for application as a point-of-care test, particularly the RPA-Cas12a-LFD assay. Notably, *N. gonorrhoeae* and *N. meningitidis* share a high degree of homology (Vigué and Eyre-Walker 2019), making it difficult to distinguish between the two pathogens during detection. The RPA-Cas12a-based *N. gonorrhoeae* detection method targeting the *porA* pseudogene had no cross-reactivity with other pathogens, and accurately distinguished *N. gonorrhoeae* from *N. meningitidis*. Therefore, its value for clinical *N. gonorrhoeae* detection is significant. In addition to its great specificity, the RPA-Cas12a system demonstrated a high sensitivity with a limit of detection (LoD) of 5 pg/µL. Besides, the practicability of the detection system was evaluated using 24 clinical samples, showing a 100% concordance with the traditional culture method. Our method promises clinical applications for the identification of *N. gonorrhoeae* due to its high feasibility.

Although the RPA-Cas12a system was successfully applied to *N. gonorrhoeae* detection, our study has several limitations. According to the TwistDx instruction manual, primer amplification performance cannot be judged only by primer sequence, candidate primers need to be tested and screened. However, we screened only six candidate primer pairs and prioritized primer specificity over sensitivity during primer screening. The RPA-Cas12a-based *N. gonorrhoeae* detection system developed in this study was less sensitive than the previously reported 1 copy/µL, possibly due to the RPA primer selection. Encouragingly, there were no false negatives when compared with the results of the gold standard traditional culture method, indicating that the sensitivity of 5 pg/µL of this method is adequate for clinical detection. Constant shaking during the RPA reaction has been shown to enhance the RPA amplification rate and improve sensitivity (Li et al. 2018), which could help to improve the LoD of the RPA-Cas12a system. To improve the sensitivity of the RPA-Cas12a system, further research should focus on primers with higher amplification efficiency and RPA reactions with constant shaking. In this study, only species identification of *N. gonorrhoeae* was carried out. With the high levels of antimicrobial resistance (AMR) (Unemo et al. 2019), further development of a test based on Cas12a that can simultaneously detect AMR, will make a great contribution to both antibiotic selection and treatment of multidrug-resistant gonorrhoea.

In conclusion, we demonstrated a promising point-of-care test for *N. gonorrhoeae* with high sensitivity and specificity based on the RPA-Cas12a detection system. Moreover, the RPA-Cas12a-LFD assay can be widely available, which may contribute significantly to *N. gonorrhoeae* management in settings with limited medical facilities.

## Supplementary Information

**Additional file 1: Table S1. **The NCBI accession numbers of *porA* gene of *N. meningitides*. **Table S2.** Primers used in this study. **Table S3.** crRNA sequence (5-3’). **Table S4.** ssDNA reporter sequence.

## Data Availability

Data available on request from the authors.
