# Supplementary material for: Point-of-care detection of Neisseria gonorrhoeae based on RPA-CRISPR/Cas12a
Source: AMB Express. 2023 May 27;13:50. doi: 10.1186/s13568-023-01554-7 (PMC10224890; doi:10.1186/s13568-023-01554-7)
Supplement: Supplementary file 1 — Additional file 1: Table S1. The NCBI accession numbers of porA gene of N. meningitides. Table S2. Primers used in this study. Table S3. crRNA sequence (5-3’). Table S4. ssDNA reporter sequence. [file 13568_2023_1554_MOESM1_ESM.docx]

Supplementary Information

Journal: AMB Express
Manuscript Title: **Point-of-care detection of *Neisseria gonorrhoeae* based on RPA-CRISPR/Cas12a**

Qianrong Tu^1^, Xiaoying Cao^2^, Chao Ling^1^, Lili Xiang^3^, Ping Yang* ^1^, Shifeng Huang*^1^

*Correspondence: Ping Yang, 201903@hospital.cqmu.edu.cn;

Shifeng Huang, [sfhuang@hospital.cqmu.edu.cn](mailto:sfhuang@hospital.cqmu.edu.cn)

^1^Department of Clinical Laboratory Medicine, the First Affiliated Hospital of Chongqing Medical University, No. 1 Friendship Road, Yuzhong District, Chongqing 400016, People’s Republic of China

^2^Department of Burn and Plastic Surgery, the First Affiliated Hospital of Chongqing Medical University, No. 1 Friendship Road, Yuzhong District, Chongqing 400016, People’s Republic of China

^3^Department of Clinical Laboratory Medicine, Chongqing Shapingba District Chenjiaqiao hospital, Chongqing 401331, People’s Republic of China

**Sequences involved in this study**

**Table S1** The NCBI accession numbers of *porA* gene of *N. meningitidis*

| AF255001.1 | KF885323.1 | KF885285.1 | KF885247.1 | DQ094070.1 | DQ094032.1 |
| --- | --- | --- | --- | --- | --- |
| AF255000.1 | KF885322.1 | KF885284.1 | KF885246.1 | DQ094069.1 | DQ094031.1 |
| KX211969.1 | KF885321.1 | KF885283.1 | KF885245.1 | DQ094068.1 | DQ094030.1 |
| KX211968.1 | KF885320.1 | KF885282.1 | KF885244.1 | DQ094067.1 | DQ094029.1 |
| KX211967.1 | KF885319.1 | KF885281.1 | KF885243.1 | DQ094066.1 | DQ094028.1 |
| AF162858.1 | KF885318.1 | KF885280.1 | KF885242.1 | DQ094065.1 | DQ094027.1 |
| AF287956.1 | KF885317.1 | KF885279.1 | KF885241.1 | DQ094064.1 | DQ094026.1 |
| KF885354.1 | KF885316.1 | KF885278.1 | KF885240.1 | DQ094063.1 | DQ094025.1 |
| KF885353.1 | KF885315.1 | KF885277.1 | KF885239.1 | DQ094062.1 | DQ094024.1 |
| KF885352.1 | KF885314.1 | KF885276.1 | KF885238.1 | DQ094061.1 | DQ094023.1 |
| KF885351.1 | KF885313.1 | KF885275.1 | KF885237.1 | DQ094060.1 | DQ094022.1 |
| KF885350.1 | KF885312.1 | KF885274.1 | KF885236.1 | DQ094059.1 | DQ094021.1 |
| KF885349.1 | KF885311.1 | KF885273.1 | KF885235.1 | DQ094058.1 | DQ094020.1 |
| KF885348.1 | KF885310.1 | KF885272.1 | KF885234.1 | DQ094057.1 | DQ094019.1 |
| KF885347.1 | KF885309.1 | KF885271.1 | KF885233.1 | DQ094056.1 | DQ094018.1 |
| KF885346.1 | KF885308.1 | KF885270.1 | KF885232.1 | DQ094055.1 | DQ094017.1 |
| KF885345.1 | KF885307.1 | KF885269.1 | KF885231.1 | DQ094054.1 | DQ094016.1 |
| KF885344.1 | KF885306.1 | KF885268.1 | KF885230.1 | DQ094053.1 | DQ094015.1 |
| KF885343.1 | KF885305.1 | KF885267.1 | KF885229.1 | DQ094052.1 | DQ094014.1 |
| KF885342.1 | KF885304.1 | KF885266.1 | KF885228.1 | DQ094051.1 | DQ094012.1 |
| KF885341.1 | KF885303.1 | KF885265.1 | KF885227.1 | DQ094050.1 | DQ094011.1 |
| KF885340.1 | KF885302.1 | KF885264.1 | AF254999.1 | DQ094049.1 | DQ094010.1 |
| KF885339.1 | KF885301.1 | KF885263.1 | AF254998.1 | DQ094048.1 | DQ092349.1 |
| KF885338.1 | KF885300.1 | KF885262.1 | AF254997.1 | DQ094047.1 | DQ092348.1 |
| KF885337.1 | KF885299.1 | KF885261.1 | AF305880.1 | DQ094046.1 | DQ092347.1 |
| KF885336.1 | KF885298.1 | KF885260.1 | AY737715.1 | DQ094045.1 | DQ092346.1 |
| KF885335.1 | KF885297.1 | KF885259.1 | DQ094082.1 | DQ094044.1 | DQ092345.1 |
| KF885334.1 | KF885296.1 | KF885258.1 | DQ094081.1 | DQ094043.1 | AY465903.1 |
| KF885333.1 | KF885295.1 | KF885257.1 | DQ094080.1 | DQ094042.1 | AY465902.1 |
| KF885332.1 | KF885294.1 | KF885256.1 | DQ094079.1 | DQ094041.1 | AF262931.1 |
| KF885331.1 | KF885293.1 | KF885255.1 | DQ094078.1 | DQ094040.1 | AF262930.1 |
| KF885330.1 | KF885292.1 | KF885254.1 | DQ094077.1 | DQ094039.1 | AF262929.1 |
| KF885329.1 | KF885291.1 | KF885253.1 | DQ094076.1 | DQ094038.1 | U94965.1 |
| KF885328.1 | KF885290.1 | KF885252.1 | DQ094075.1 | DQ094037.1 | U94964.1 |
| KF885327.1 | KF885289.1 | KF885251.1 | DQ094074.1 | DQ094036.1 | U94959.1 |
| KF885326.1 | KF885288.1 | KF885250.1 | DQ094073.1 | DQ094035.1 | U94958.1 |
| KF885325.1 | KF885287.1 | KF885249.1 | DQ094072.1 | DQ094034.1 | AJ007407.1 |
| KF885324.1 | KF885286.1 | KF885248.1 | DQ094071.1 | DQ094033.1 | AJ012189.1 |

**Table S2** Primers used in this study

| RPA | Primers | Sequence (5’-3’) |
| --- | --- | --- |
|  | Primer pair 1 |  |
|  | F | CCACGACGGTATGCCGGTTTCCGTGCGTTA |
|  | R | GAACTGGTTTCATCTGATTACTTTCCAGCGT |
|  | Primer pair 2 |  |
|  | F | ATTGGGTATTTTCAAACGCCACGACGGTAT |
|  | R | GAACTGGTTTCATCTGATTACTTTCCAGCGT |
|  | Primer pair 3 |  |
|  | F | GCTCGCCGGTCGCGTTGCGAATCCGTTTGG |
|  | R | GAACTGGTTTCATCTGATTACTTTCCAGCGTG |
|  | Primer pair 4 |  |
|  | F | CAAAGCCATTGATCCTTGGGACAGCAATAATA |
|  | R | GAACTGGTTTCATCTGATTACTTTCCAGCGTG |
|  | Primer pair 5 |  |
|  | F | ATCGGCTTGGCAGGCGAATTCGGCACGGCGCT |
|  | R | GAACTGGTTTCATCTGATTACTTTCCAGCGTG |
|  | Primer pair 6 |  |
|  | F | CAATAATAATGTGGCTTCGCAATTGGGTATT |
|  | R | CGGAACTGGTTTCATCTGATTACTTTCCAG |

**Table S3** crRNA sequence (5’-3’)

| crRNA | UAAUUUCUACUAAGUGUAGAUAGCGGCAGCAUUCAAUUUGUU |
| --- | --- |

**Table S4** ssDNA reporter sequence

| Fluorescent reporter | 5’-/6-FAM/CCGGAAAAAAAAAAAACCGG/BHQ1/-3’ |
| --- | --- |
| Lateral flow reporter | 5’-/6-FAM/TTATTATT/Biotin/-3’ |
